# “What if someone reads these records and judges me?”: fear, legitimacy, and the hidden curriculum in veterinary clinical decision-making

**DOI:** 10.3389/fvets.2026.1958601

**Published:** 2026-09-25

**Authors:** Sara Ritzie, Jason Coe, Elizabeth C. Kurucz, Lynn Henderson, Andria Jones, Lauren E. Van Patter, Shane Bateman

**Affiliations:** 1 Ontario Veterinary College, Department of Clinical Studies, University of Guelph, Guelph, ON, Canada; 2 Ontario Veterinary College, Department of Population Medicine, University of Guelph, Guelph, ON, Canada; 3 Gordon S. Lang School of Business and Economics, University of Guelph, Guelph, ON, Canada

**Keywords:** clinical decision-making, contextualized care, hidden curriculum, moral distress, professional identity, spectrum of care, veterinary culture

## Abstract

**Introduction:**

Spectrum of Care (SpOC) has been promoted as a means of improving access to veterinary care while supporting ethical and sustainable clinical decision-making. Yet many veterinarians who value contextualized, client-centered care continue to experience difficulty incorporating SpOC approaches into everyday practice. While these challenges are often framed as deficits in knowledge, resources, or training, less attention has been paid to the influence of professional culture.

**Methods:**

This qualitative study drew on data collected as part of a mixed-methods evaluation of a SpOC workshop delivered to veterinarians in Ontario, Canada. Data sources included semi-structured interviews conducted 3 months following workshop participation (*n* = 17) and open-text questionnaire responses collected before, immediately after, and 3 months following the intervention. Data were analyzed using reflexive thematic analysis.

**Results:**

Participants described numerous strategies that supported contextualized care, including collaborative communication, shared decision-making, supportive team cultures, knowledge of alternative care pathways, and a growth-oriented mindset. However, fear emerged as a pervasive influence on clinical decision-making. Veterinarians described concerns about peer judgment, client complaints, regulatory scrutiny, and the possibility that context-appropriate decisions would later be interpreted as substandard care. Importantly, these concerns often reflected anticipated rather than experienced criticism. Participants linked these fears to implicit professional norms learned through veterinary training and reinforced throughout practice, describing a hidden curriculum that privileges idealized or gold-standard approaches, narrows perceptions of acceptable care, and creates pressure to conform even when alternative approaches are clinically appropriate.

**Discussion:**

These findings suggest that contextualized care may be constrained less by deficiencies in clinical knowledge than by professional and cultural forces that shape perceptions of legitimate care. Fear emerged as an immediate influence on decision-making, while the hidden curriculum functioned as a potential underlying mechanism, generating and sustaining that fear. These findings position SpOC not only as a clinical framework for improving access to care, but also as a lens through which broader questions of professional culture, legitimacy, and veterinary identity become visible. Until contextualized care is culturally legitimized alongside idealized models of care, fear and the hidden curriculum may continue to undermine efforts to improve access to care and support practitioner wellbeing.

## 1 Introduction

Access to veterinary care has emerged as a significant challenge in companion animal medicine (1–5). While similar challenges are reported globally, current literature disproportionately reflects North American contexts, and recent studies suggest these barriers have intensified in recent years (6). Financial, geographic, and social barriers prevent many families from obtaining veterinary services, with consequences for animal welfare, client wellbeing, and veterinary professionals who must regularly navigate constrained care decisions (1, 5, 7–11). In parallel, veterinary professionals face rising client expectations, economic pressures, and evolving standards of care (12). These factors contribute to moral distress and other negative wellbeing outcomes (13–21), particularly when practitioners feel unable to provide care they believe is in their patients’ best interests (8, 15, 22–27). A previous analysis of data from this study identified access-to-care constraints as a common source of ethical dilemmas, emphasizing the close relationship between barriers to veterinary care and practitioner wellbeing (28).

SpOC can provide a framework for navigating some of these challenges by encouraging veterinarians to consider and offer evidence-based diagnostic and treatment options that vary in complexity, cost, invasiveness, and resource requirements (12, 29–35). Rather than viewing veterinary care as a single ideal pathway, SpOC recognizes that multiple medically acceptable approaches may exist and that the most appropriate option depends on the patient, client, and clinical setting (34). While discussions of SpOC are often linked to affordability, context-sensitive decision-making may also arise from considerations related to patient welfare, prognosis, quality of life, owner values, geographic limitations, treatment burden, or the relative benefits and burdens of continued treatment, including euthanasia. Closely related to this concept is contextualized care, which involves tailoring these options to the circumstances, values, and constraints of individual clients and patients (30). Throughout this paper, SpOC is conceptualized as a *framework* that recognizes multiple acceptable diagnostic and treatment pathways, whereas contextualized care describes the *process* of tailoring those options to individual circumstances.

SpOC has been proposed as a means of supporting flexible, context-appropriate decision-making to benefit clients, patients, and veterinary professionals (8, 27, 29, 36–41). Veterinary organizations and educational institutions have increasingly recognized its importance (42–48). However, many practitioners continue to report difficulty incorporating SpOC principles into routine clinical decision-making (38, 49).

Existing discussions of challenges in practicing SpOC often focus on practical considerations such as limited time, financial constraints, insufficient evidence, and a lack of familiarity with alternative approaches (29, 33, 34, 49). While these factors are important, a growing body of literature suggests that professional culture may also shape how practitioners perceive and deliver care (13, 14, 26, 30, 38, 50–56). Fear of complaints, regulatory scrutiny, peer judgment, and adverse patient outcomes can influence clinical decision-making and encourage more defensive approaches to practice (21, 29, 40, 49, 51, 54, 57–63). Such pressures may discourage flexibility and make veterinarians hesitant to offer options that deviate from perceived norms, directly undermining the foundations of contextualized care and representing a substantial barrier to adopting a SpOC model.

Importantly, these influences do not necessarily depend on overt criticism or formal sanction. Perceptions of what colleagues, employers, specialists, regulators, or the profession more broadly may view as acceptable can shape behaviour even in the absence of direct feedback (64). As a result, veterinary clinical decision-making occurs not only within a clinical context but also within a social and professional context, where judgments of competence, appropriateness, and quality of care are continually negotiated.

These pressures may be reinforced through what has been described as a hidden curriculum of veterinary medicine: the informal set of norms, values, and expectations that permeate the profession as a whole, subtly shaping professional identity, clinical decision-making, and definitions of success (31, 55, 65). The hidden curriculum is thought to shape beliefs about what constitutes good medicine, professional competence, and acceptable care (55). Within veterinary medicine, it has been associated with an emphasis on idealized or *gold-standard* care, specialization, technical expertise, and perfectionism (53, 55, 66). Although the term lacks a universally accepted definition, *gold standard* is commonly used to describe the most comprehensive, or resource-intensive diagnostic and treatment approach available, often informed by specialist expertise and current scientific evidence (29, 30). These norms are generally intended to promote excellence; however, they may inadvertently limit adaptability, stigmatize alternative care pathways, and contribute to feelings of inadequacy when veterinarians are unable to pursue idealized recommendations (30, 31, 34, 52, 67, 68). Consequently, practitioners may experience tension between contextualized care and prevailing professional expectations regarding what forms of care are viewed as legitimate.

Despite increased discussion of the hidden curriculum in veterinary education and growing interest in SpOC, relatively little empirical research has examined how practicing veterinarians experience these influences in clinical practice. In particular, little is known about how practitioners navigate tensions between professional expectations, personal values, client realities, and perceptions of acceptable care. These experiences may be shaped not only by clinical or logistical barriers, but also by cultural and professional factors that influence how veterinarians define, justify, and evaluate their decisions.

This study draws on qualitative data collected during a mixed-methods evaluation of a SpOC workshop for practicing veterinarians. While the original study focused on workshop experiences and outcomes, participants consistently described broader influences affecting their ability to provide contextualized care. This article explores those influences, with particular attention to fear, professional culture, and the hidden curriculum. In doing so, it contributes to a deeper understanding of the cultural and social forces shaping veterinary practice.

The objective of this study was to examine how veterinarians navigate contextualized care within the social, professional, and organizational contexts of practice. Drawing on qualitative findings from a mixed-methods evaluation of a SpOC workshop, our analysis sought to move beyond practical implementation challenges to explore the cultural forces shaping perceptions of acceptable care.

Specifically, this study aimed to:

1) Understand how veterinarians create opportunities for contextualized care through communication, team culture, knowledge development, and reflective practice.
2) Explore the role of fear-including concerns about complaints, professional judgment, and regulatory repercussions-in shaping clinical decision-making related to contextualized care.
3) Examine the influence of the veterinary hidden curriculum on veterinarians’ perceptions of legitimate care and their willingness to depart from idealized models of practice.

## 2 Methods

This manuscript presents a secondary qualitative analysis of data collected during a mixed-methods evaluation of a SpOC workshop for veterinarians (69). The broader study combined qualitative interviews and questionnaire responses with quantitative measures to examine perceived workshop impacts on confidence, wellbeing, and the ability to practice SpOC. In the qualitative dataset, participants repeatedly described broader influences on clinical decision-making and contextualized care which extended beyond the original intervention-focused objectives and prompted further exploration of the role of professional culture, fear, and the hidden curriculum in veterinary practice, which form the focus of the present analysis.

### 2.1 Participant recruitment and sampling

Participants were recruited through professional veterinary networks, email distribution lists, and social media with support from several veterinary organizations. Eligible participants were licensed veterinarians working in general practice. Twenty-two veterinarians participated in the broader study. Participants represented a range of career stages, practice settings, and geographic locations. Most reported primarily working in companion animal practice, and the majority had more than 10 years of clinical experience. Respondents were predominantly women and represented both urban and rural practice settings. Because questionnaire responses were anonymous, demographic information could not be linked to interview participants.

### 2.2 Workshop description

The workshop was designed to support veterinarians in applying SpOC principles in practice. Content included contextualized care, communication and shared decision-making strategies, and clinical decision-support tools. Learning activities incorporated reflective practice and facilitated group discussion.

### 2.3 Data collection

Qualitative data consisted of semi-structured interviews conducted approximately 3 months after workshop participation and open-text questionnaire responses collected before the workshop (T1), immediately afterward (T2), and 3 months later (T3) (70). These time points were selected as part of the broader mixed-methods evaluation to capture participants’ baseline perspectives, immediate reactions to the workshop, and experiences applying workshop concepts in practice. Interviews followed a semi-structured guide developed for the broader workshop evaluation (Table 1). They were conducted, recorded, and automatically transcribed via Microsoft Teams (Microsoft Corporation, Redmond, United States, 2025) (71), and reviewed for accuracy by the first author. Questionnaires were administered in English using Qualtrics (Qualtrics, Provo, UT, United States July, 2025) (72). Only qualitative data sources were included in the present analysis.

**Table 1:** Semi-structured interview guide.

Ice-breaker questions: How long have you been in practice? What do you love about practice? What has surprised you about practice?
In your opinion, who are the stakeholders involved in addressing client barriers to accessing veterinary care and what are their roles/responsibilities? What role do you play in the face of client barriers to accessing veterinary care? What challenges do you face when dealing with barriers to accessing veterinary care?
What (if any) changes have you made or actions have you taken in terms of Spectrum of Care since participating in the workshop? What has been the impact of these implemented changes? In the past 3 months, how many cases visits have you used a spectrum of care approach with? How do you approach sharing the spectrum of options with clients? Thinking about the range of options presented to the client, where along the range was selected (low/middle/high)? What were the patient outcomes? What aspects of the SoC approach taken in these case(s) were viewed positively by the veterinary team? What aspects of the SoC approach taken in these case(s) were viewed negatively by the veterinary team?
What (if any) additional training and/or resources would help enable you to increase the spectrum of care you practice?

### 2.4 Data analysis

Qualitative data were analyzed using reflexive thematic analysis following the six-phase approach described by Braun and Clarke: familiarization with the data, initial coding, theme development, theme review, theme refinement, and narrative construction (73). Analysis was inductive and data-driven, with themes developed through iterative engagement with participant accounts rather than a predetermined framework.

Interview transcripts and questionnaire responses were imported into standard software (NVivo 14, Release 14.24.3, Denver, United States, 2023) (74) and analyzed as a single qualitative dataset to facilitate data organization and coding. Coding was conducted by the first author and refined iteratively through repeated engagement with the dataset and ongoing analytic reflection. Themes were developed through comparisons across interviews and questionnaire responses, with attention to recurrent patterns in contextualized care, clinical decision-making, professional expectations, and veterinary culture.

To enhance rigor, reflexive notes were maintained throughout analysis as a form of memoing (75). Peer debriefing was conducted with two co-authors (LVP & AJ) who reviewed selected transcripts and coding decisions. Participants were also provided with copies of their interview transcripts and invited to offer clarifications or comments.

The primary researcher (SR) served as workshop developer, facilitator, interviewer, and analyst. As a practicing veterinarian with an interest in SpOC and access to care, SR’s perspectives inevitably influenced data generation and interpretation, as consistent with reflexive qualitative methodology. These influences were acknowledged and critically examined throughout the research process (76–79). This study adopts the view that knowledge is co-constructed between researchers and participants and seeks to generate an interpretive understanding of how veterinarians experience contextualized care within the professional, organizational, and cultural contexts of practice.

## 3 Results

Two workshops were delivered in August 2024. A total of twenty-two veterinarians attended the workshops and provided qualitative questionnaire data. Of these, seventeen participated in follow-up interviews approximately 3 months later. Data sufficiency was judged to have been reached before all interviews were coded, with later interviews contributing depth and variation rather than substantially new concepts (80). Three interconnected themes were created: (1) Creating space for contextualized care, (2) Fear and clinical decision-making, and (3) The hidden curriculum and perceptions of acceptable care (Figure 1).

**Figure 1:**
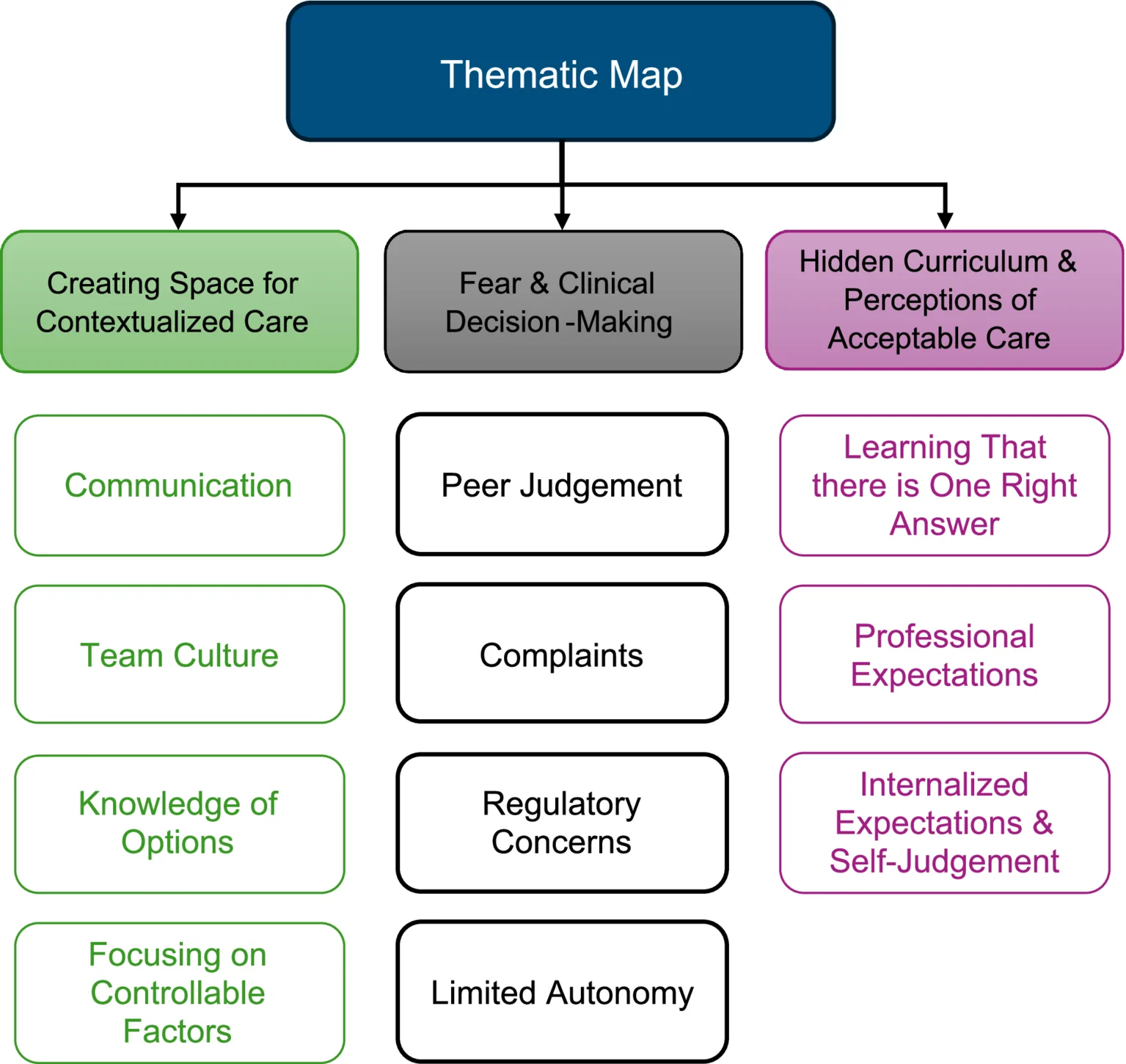
Thematic map of factors influencing contextualized care. Thematic map illustrating the three interconnected themes identified through qualitative analysis and their associated subthemes. The figure summarizes how participants described creating space for contextualized care, the influence of fear on clinical decision-making, and the role of the hidden curriculum in shaping perceptions of acceptable care.

### 3.1 Creating space for contextualized care

Participants described a range of strategies that enabled them to provide care that better reflected client circumstances and patient needs. These included communication, team culture, knowledge development, and reflective practice, all of which were described as creating space for contextualized care within the constraints of everyday practice. These strategies involved both outward-facing actions directed toward clients and teams, as well as internal processes related to learning, self-reflection, and mindset.

#### 3.1.1 Communication and shared decision-making

Every participant identified *communication* as central to practicing SpOC. Participants emphasized the importance of exploring clients’ circumstances, understanding barriers to care, and creating space for collaborative discussions about care options. Rather than making assumptions about what clients could or could not pursue, veterinarians described using open-ended conversations to better understand client needs and priorities. One participant reflected:

*“I think we kinda have to be willing and able to be flexible or investigate, like even just scratch the surface a little bit and find out, OK why?” (P1).*

Participants also described moving away from a paternalistic decision-making model toward a more collaborative approach in which clients were supported in selecting among medically appropriate options.

*“I am not trying to make decisions for families but trying to offer them guidance and recommendations as they navigate through their pet’s health–like a team-based approach.” (P4).*

Communication was therefore viewed not simply as a means of conveying information, but as a process through which veterinarians and clients collaboratively construct context-appropriate care plans.

#### 3.1.2 Building supportive team cultures

Participants consistently described clinic culture as influencing whether contextualized care felt possible within practice. Supportive teams were characterized by openness, reflection, and alignment around shared goals of helping clients and patients navigate challenging circumstances.

Several participants spoke about team culture not only as an organizational feature, but also as something actively shaped through day-to-day interactions and leadership behaviours. Veterinarians identified themselves as leaders who help shape the clinic culture through their behaviour and expectations.

*"Be sure to try to always model the best behaviour because staff was very quick to pick up on what was coming from the top."* (P18)

Participants described practices such as regular team discussions as important ways to identify assumptions and barriers that might inadvertently limit access to care.

*"Repetitive checking in between the team even can be very useful in terms of how we’re communicating with clients and knowing what’s going on … and bringing awareness to our own perceptions and obstacles that we're putting on ourselves."* (P8)

Participants suggested that cultures that support flexibility, reflection, and collaboration enable veterinarians to explore a wider range of care options with confidence.

#### 3.1.3 Building knowledge and confidence

Participants reported that confidence in offering context-appropriate care requires familiarity with multiple diagnostic and treatment pathways. Although participants generally supported offering a range of options, many expressed uncertainties with alternative approaches due to limited exposure, evidence, and training. Some participants expressed a desire for additional outcomes data to support recommendations that departed from conventional pathways.

*"If we have data that says, “We know that if we offer more moderate care options, we’re gonna have higher uptake”, then that’s easier to get behind … I think we need the data to help us get over that hurdle, to help us understand."* (P18)

Resources outlining alternative approaches were described as particularly valuable. For example:

*“I really appreciated the resources that said, ‘Well, if you can’t do this, how about protocol B?’ And like, that protocol B is awesome to have.”* (P3)

Participants viewed knowledge of feasible alternatives as important for increasing confidence when discussing recommendations that differed from more conventional approaches.

#### 3.1.4 Focusing on controllable factors

Alongside practical strategies, participants described internal shifts that helped them navigate the emotional and ethical complexity of clinical practice. Many emphasized focusing on what was within their control while accepting the realities and constraints present in veterinary medicine.

*“This is the piece that I can control and exist in. And I can only make the changes and the difference that I can make as a human … but that’s OK.”* (P9)

Others described the importance of self-compassion when ideal outcomes were unattainable.

*“You do what time and resources allow … And the key part … is that after you’ve done the best you can, you forgive yourself and try to let it go.”* (P5)

Collectively, these strategies helped participants create conditions that supported contextualized care despite external constraints.

### 3.2 Fear as a social influence on clinical decision-making

Although participants described ways of creating space for SpOC, they also identified fear as a persistent influence on clinical decision-making. Fear was discussed across diverse contexts, including concerns about peers, employers, clients, regulators, and future evaluation of clinical choices.

#### 3.2.1 Fear of professional judgment

Participants frequently expressed concern that clinical decisions would be negatively evaluated by others, even when those decisions reflected careful consideration of the situation.

*“I think a lot of that comes down to, like, not doubting that you feel that’s right for your patient, but that someone else is going to look at that and say that it’s not right.”* (P9)

Notably, participants frequently described anticipating judgment rather than experiencing it directly. Several participants described lingering concerns about how records or decisions might be interpreted in the future.

*“What if someone reads these medical records in the future and they judge me?”* (P6)

These fears created hesitation around offering alternatives that deviated from perceived professional expectations.

#### 3.2.2 Fear of complaints and regulatory consequences

Concerns regarding complaints and professional oversight emerged repeatedly throughout interviews. Participants questioned whether context-sensitive decisions would be supported if challenged by clients, colleagues, or regulators.

*“There’s … anxiety about what if, what if we didn't do everything that we could have and we missed something and it kind of comes back to haunt me, whether that’s a [licensing body] complaint or social media or another doctor judging you.”* (P2)

Even participants aware of regulatory statements supporting a SpOC approach remained uncertain about how those principles would be applied in the event of adverse outcomes or complaints.

*“I don't feel protected by explaining to owners … I've seen the vet still hang out to dry and I find that, feeling that that could happen is a big frustration.”* (P11)

*“I still sometimes see situations where a veterinarian’s been punished—suspended, fined, sometimes a lot of money. And I can read between the lines that something just didn’t go well … If we’ve done our due diligence, I don’t think it’s fair for a veterinarian to be punished in that way for not doing better medicine when they’re really just between a rock and a hard place, trying to help.”* (P3)

For many participants, concerns about complaints and scrutiny led them to be cautious when considering flexible care approaches.

#### 3.2.3 Limited autonomy

Participants also described constraints on their ability to provide contextual care, particularly when working in settings perceived as highly structured or hierarchical. While financial policies were one source of frustration, participants more broadly described limited flexibility in tailoring care plans or responding to client circumstances.

Several participants working in corporate settings described feeling caught between supporting clients and fulfilling organizational expectations:

*“My clinic is a corporate clinic—so it feels like an even bigger, more kind of daunting authority … you're kind of a middleman between being a friend and a helper and a guide for the client, but also making sure you're adhering to your responsibilities as an employee.”* (P12)

Others described environments that limited their ability to implement changes or adapt care approaches:

*“I haven't had the opportunity to implement or to make a lot of changes … we're a little bit more strict and rigid the way we conduct things here.”* (P16)

One participant contrasted these experiences with previous practice settings that afforded greater flexibility:

*“Private practice in a rural area felt more like a community with clients, like you were trying to help out your neighbors … With corporate practice, you still have a community but it’s different. It feels less neighbourly and more administrative. I hear clients struggling with cost more and options are bit more restrictive.”* (P4)

These accounts suggest that organizational structures may influence not only the options available to clients, but also veterinarians’ perceived ability to exercise professional judgment. In some cases, organizational constraints interacted with fears of scrutiny or criticism, leaving participants feeling unable to act on their own contextualized clinical judgment.

### 3.3 The hidden curriculum and perceptions of legitimate care

Rather than emerging in isolation, fear was often described as a response to broader professional expectations learned through veterinary training and reinforced throughout practice. These accounts reflected characteristics of a hidden curriculum that shaped perceptions of competence, legitimacy, and acceptable care.

#### 3.3.1 Learning that there is one right answer

Many participants described educational and professional experiences that emphasized idealized approaches to care while providing limited exposure to navigating practical constraints or discussing alternatives. Several participants reported that veterinary training focused heavily on identifying the correct diagnostic or treatment pathway, with comparatively little attention given to adapting care to individual circumstances.

*“We never discussed in school, “options” … So when I came out, there was a lot of like, whoa … now I gotta be creative. So I think in school is the start point for sure.”* (P2)

Others suggested that training environments often failed to reflect the realities of general practice.

*“We teach that the world is not perfect, but then we teach medicine like it is.”* (P3)

Participants frequently linked these experiences to the profession’s emphasis on *gold standard* care. While these expectations were intended to promote excellence, participants described them as narrowing perceptions of acceptable care and creating pressure to pursue idealized pathways regardless of context. Several participants suggested that the issue was not whether alternative approaches could be clinically appropriate, but whether they would be viewed as professionally acceptable.

*“I was taught and browbeaten about gold-standard care. We were trained like, if you don’t do it this way, it’s gonna turn out horribly … which is not true.”* (P18)

Participants repeatedly described trying to practice contextualized care while navigating a professional culture that prioritized idealized approaches and made deviation from those approaches feel risky. These experiences contributed to reluctance around offering lower-cost, less invasive, or alternative pathways, even when participants believed those approaches were reasonable and clinically appropriate for a particular patient and client.

#### 3.3.2 Internalized expectations and self-judgment

Perhaps most strikingly, participants described how professional expectations became internalized and self-reinforcing. The fear of judgment was frequently described as originating within the profession rather than exclusively from external stakeholders.

One participant summarized this sentiment:

*“We judge ourselves and each other. It's not just the public. It's not the [licensing body]. It's not social media, it's us.”* (P2)

This observation captures the central tension evident in the dataset: participants valued contextualized care and actively sought ways to implement it, yet they frequently expressed concerns that deviating from idealized models of care could invite judgment or criticism. Participants linked these concerns to professional norms learned through training and reinforced throughout practice, suggesting that perceptions of acceptable care extended beyond individual clinical choices.

## 4 Discussion

This study explored how veterinarians navigate contextualized care using a SpOC framework within the social, professional, and organizational contexts of practice. Participants described both deliberate strategies that helped them provide context-appropriate care and substantial barriers that constrained their ability to do so. While communication skills, supportive team cultures, knowledge of alternative care pathways, and reflective mindsets were identified as important enablers, fear emerged as a pervasive challenge. Participants frequently linked this fear to broader professional norms and expectations, suggesting that contextualized decision-making may be shaped as much by veterinary culture as by clinical knowledge or technical capability. Building on the thematic findings presented in Figures 1, 2 provides a conceptual synthesis of the relationships among professional culture, fear and uncertainty, perceptions of legitimate care, and contextualized decision-making. Taken together, the findings suggest that efforts to expand contextualized care require not only practical skills and resources, but also professional cultures that legitimize flexible, context-sensitive care (Figure 2).

**Figure 2:**
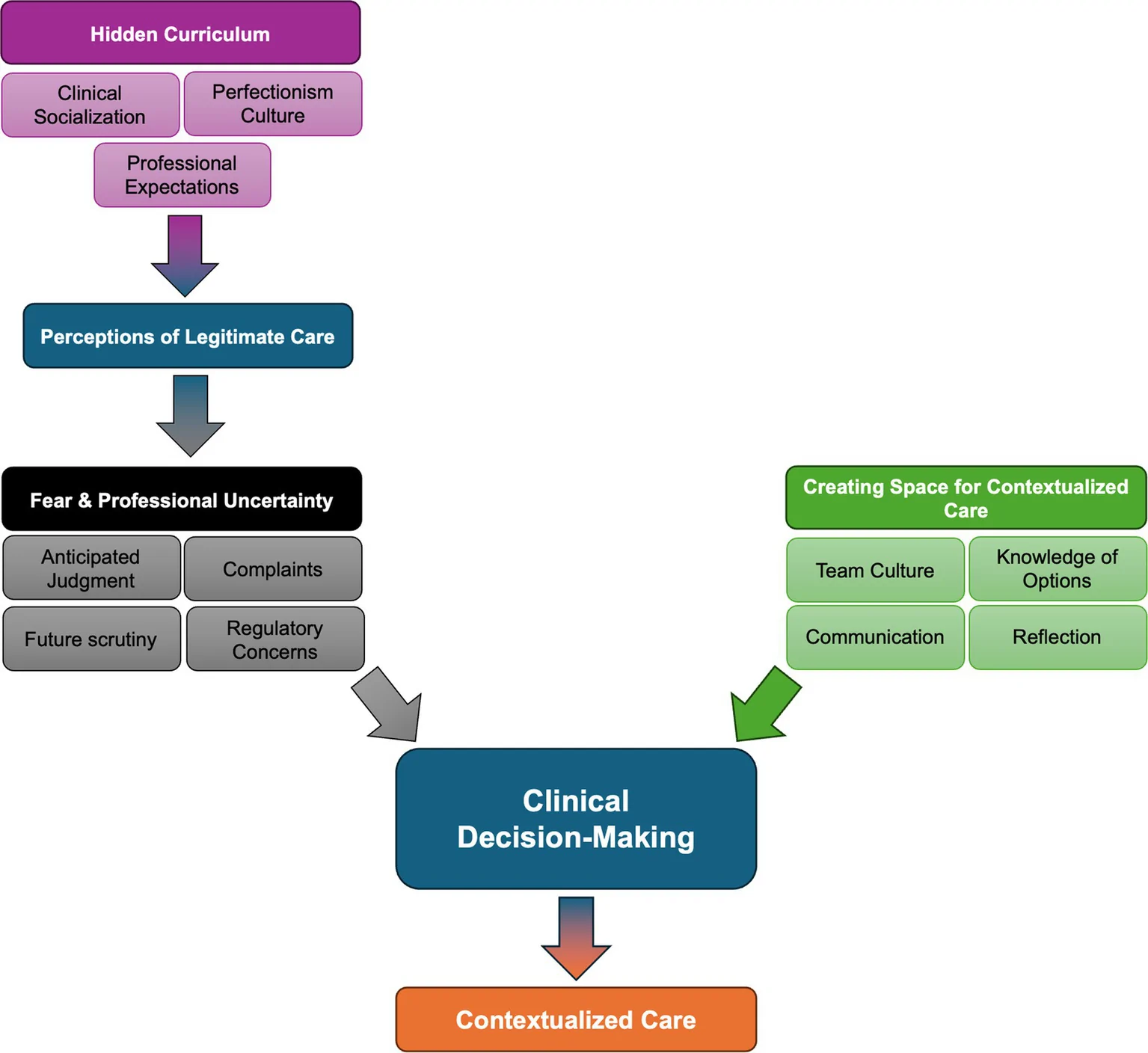
Conceptual model of contextualized care decision-making in veterinary practice. Conceptual synthesis of the three themes identified through qualitative analysis, illustrating how participants described relationships among the hidden curriculum, fear and professional uncertainty, and practices that create space for contextualized care. The model is intended as a knowledge-translation framework that summarizes relationships among themes rather than as a causal or predictive model.

### 4.1 Veterinarians actively create opportunities for contextualized care

Participants described numerous ways of expanding access to care within the constraints of everyday practice. Communication and shared decision-making (81, 82) were consistently identified as essential components of contextualized care, reinforcing previous research demonstrating that collaborative approaches strengthen trust, improve client engagement, and support care aligned with client circumstances and values (31, 40, 81, 83, 84). Rather than viewing clients as passive recipients of recommendations, participants described intentionally exploring client goals, limitations, and priorities to identify feasible pathways forward. These findings align with growing calls within veterinary medicine to move away from paternalistic models of decision-making toward more collaborative, relationship-centered approaches to care (27, 40, 41, 82, 85, 86).

Supportive team culture also emerged as an important facilitator. Participants frequently described clinics as social environments that either enabled or constrained flexibility in clinical decision-making. Practices characterized by collaboration, psychological safety, and shared commitment to helping clients navigate challenges appeared to foster greater confidence in offering a range of care options. This finding is consistent with the broader veterinary literature, which demonstrates that team culture influences both the quality of care and practitioner wellbeing (13, 87, 88). Importantly, participants often viewed themselves as active contributors to clinic culture, suggesting that contextualized care is shaped not only at the level of individual clinicians but also through collective norms and behaviours within veterinary teams.

Participants also emphasized the importance of knowledge development and self-reflection. Access to resources outlining alternative diagnostic and treatment pathways appeared to increase confidence in discussing options that departed from conventional approaches. Difficulty identifying viable care options reflected broader gaps in accessible, evidence-based resources, as documented in the literature (29, 34, 49).

At the same time, participants emphasized the importance of focusing on controllable factors. Participants described consciously directing their attention toward aspects of care they could influence while accepting the realities and constraints that often accompany veterinary practice. Reflective practice, self-compassion, and acceptance of uncertainty were frequently discussed as ways of managing the emotional burden associated with imperfect outcomes and constrained decision-making. Similar strategies have been identified in previous research on resilience-building among veterinary professionals (37, 89). These findings suggest that offering contextualized care depends not only on clinical competence but also on veterinarians’ ability to navigate uncertainty, tolerate ambiguity, and remain open to multiple context-appropriate approaches to care.

Collectively, these findings challenge the notion that barriers to SpOC arise solely from insufficient knowledge or resources. Participants demonstrated strong motivation to expand access to care and described actively developing skills and strategies to achieve this. This raises an important question: if many veterinarians value and actively pursue contextualized care, why do these approaches continue to feel difficult or risky in practice?

### 4.2 Fear as a social influence on clinical decision-making

Across interviews, fear emerged as a pervasive influence on veterinarians’ ability to practice contextualized care. Participants described feeling vulnerable to peer judgment, client complaints, regulatory scrutiny, adverse outcomes, and future retrospective evaluation of their clinical decisions. These concerns frequently surfaced when discussing situations in which context-appropriate care differed from what participants perceived to be the expected or preferred pathway.

The pervasiveness of these fears is consistent with the literature on the emotional impact of client complaints, disciplinary investigations, and professional criticism in veterinary medicine (54, 57, 61, 63, 90). Prior studies have shown that concerns about complaints can contribute to defensive medicine, where diagnostic or treatment decisions are shaped partly by practitioners’ desire to reduce personal risk rather than benefit the patient (51, 54, 57, 91). Participants’ accounts suggest that similar dynamics may influence contextualized decision-making. Although they did not explicitly label their actions as defensive, many described pressure to conform to perceived professional norms, particularly expectations for *gold standard* care. Offering anything less was seen as clinically questionable, potentially inviting criticism from colleagues or regulatory scrutiny.

Importantly, fear was rarely described as an isolated individual characteristic. Rather, participants portrayed it as a response to broader social and professional pressures. Concerns about judgment frequently extended beyond clients to include colleagues, employers, specialists, and regulatory bodies. This finding suggests that fear may be understood less as a personal barrier and more as a symptom of the environments in which veterinarians practice. In this context, efforts aimed solely at improving individual confidence or resilience may be insufficient if the broader systems that generate fear remain unchanged.

The findings, therefore, support the interpretation that fear functions as a proximal barrier to contextualized care. It is the immediate experience that shapes clinical behaviour and decision-making. However, participants’ reflections suggest that understanding fear alone does not fully explain the challenges associated with SpOC. Instead, fear appears embedded within broader professional narratives about what constitutes legitimate veterinary care.

### 4.3 The hidden curriculum as an underlying mechanism

Perhaps the most striking finding of this study was the extent to which participants connected challenges associated with contextualized care to implicit professional norms learned during training and reinforced throughout practice. Participants repeatedly described educational and professional experiences that emphasized idealized approaches to care, reinforced the perception of a single correct clinical pathway, and privileged technical excellence over contextual adaptation. These accounts closely resemble descriptions of the hidden curriculum in veterinary education literature (68, 92). Previous scholarship has argued that the hidden curriculum shapes professional identity by communicating implicit values and expectations about success, competence, and acceptable practice (31, 52). Participants’ experiences suggest that these influences may extend beyond education and continue throughout professional life. The recurring use of terms such as *gold standard*, even as participants simultaneously critiqued such language, illustrates how deeply embedded these norms are in veterinary culture. For many participants, these expectations appeared to create tension between what they believed was appropriate for a particular patient and what they perceived others would regard as acceptable care.

Notably, participants did not describe the hidden curriculum as operating primarily through explicit rules or policies. Instead, it appeared to function through social expectations, subtle judgments, and internalized beliefs about professional competence. The observation that “we judge ourselves and each other” captures how professional norms become self-reinforcing. In this sense, the hidden curriculum may represent one mechanism through which fear is generated and sustained. If veterinarians are socialized to equate good practice with idealized or resource-intensive approaches (30, 31, 34, 52), then deviation from those approaches may feel professionally risky, even when alternative options better align with client circumstances and patient needs. Other participants described feelings of inadequacy, noting that although they felt technically capable of performing certain procedures, they hesitated to do so without specialist credentials, reflecting a hierarchy embedded in veterinary culture.

These interpretations extend existing discussions of SpOC and contextualized care in veterinary practice. While previous work has focused on educational gaps, communication skills, or resource limitations, the present findings suggest that cultural legitimacy may be equally important. Participants’ accounts indicate that the hidden curriculum reinforces the perception that there is a preferred answer, a preferred diagnostic pathway, a preferred treatment plan, and a preferred professional identity against which alternative approaches are evaluated. Veterinarians may possess the knowledge and motivation required to practice contextualized care yet remain hesitant to do so if professional culture continues to frame such decisions as inferior, risky, or difficult to defend. Efforts to normalize SpOC may therefore require not only clinical tools and training but also deliberate examination of the cultural assumptions that shape perceptions of quality care.

### 4.4 Implications for veterinary education and practice

These findings have implications for veterinary education, continuing professional development, and clinical leadership. Educational initiatives focused on SpOC should extend beyond teaching alternative care pathways and communication frameworks to include critical reflection on professional norms, perfectionism, and the hidden curriculum. Creating opportunities for learners and practitioners to openly discuss uncertainty, contextual constraints, and multiple acceptable approaches to care may help reduce the stigma associated with deviating from idealized models.

At the practice level, fostering psychological safety and supportive team cultures may be equally important. Participants described clinic environments as key influences on whether contextualized care felt acceptable, suggesting that leaders play a critical role in shaping local norms and expectations. Professional organizations and regulatory bodies can also contribute by providing clearer signals that context-sensitive decision-making is both legitimate and professionally defensible when grounded in sound clinical reasoning and effective communication.

Collaboration among educators, practitioners, professional organizations, and regulatory bodies may help foster greater confidence in contextualized care.

## 5 Limitations

Several limitations should be considered when interpreting these findings. Participants were self-selected veterinarians who chose to attend a workshop focused on SpOC, and many may have already been interested in contextualized approaches to practice. As a result, the findings are not reflective of the experiences or perspectives of all veterinarians. The study was also conducted within a specific geographic and professional context, limiting transferability to other settings. In addition, the themes reported here emerged from a secondary analysis of qualitative data collected within a broader mixed-methods study. While this allowed unexpected insights to be explored, the original interview guide was not specifically designed to investigate fear, professional culture, or the hidden curriculum in depth. More focused inquiry may have yielded a richer understanding of how participants conceptualized and experienced these phenomena.

## 6 Conclusion

Veterinarians may already possess many of the skills and motivations needed to offer contextualized care, with participants describing numerous strategies that supported context-sensitive decision-making, including communication, collaboration, knowledge development, and reflective practice. However, these efforts frequently occurred within a professional environment characterized by fear of judgment, complaints, and deviation from perceived norms. The findings suggest that fear can function as an immediate barrier to contextualized care, while the hidden curriculum can serve as an underlying cultural mechanism that reinforces those fears. Advancing SpOC may therefore require more than expanding clinical skills and resources; it may require a broader cultural shift that recognizes context-sensitive care as a legitimate expression of high-quality veterinary medicine rather than a departure from it. More broadly, these findings suggest that clinical decision-making is shaped not only by medical knowledge and patient needs, but also by the professional cultures through which veterinarians learn what forms of care are considered legitimate.

## Data Availability

The datasets generated and analyzed during the current study cannot be made available by the authors because participant consent for data sharing was not obtained and the qualitative data contain information that could compromise participant confidentiality. Further inquiries can be directed to the corresponding author/s.
